# New Appliances and Things Medical

**Published:** 1900-10-06

**Authors:** 


### NEW APPLIANCES AND THINGS MEDICAL.

[We shall be glad to receive, at our Office, 28 & 29 Southampton Street, Strand, London, W.C., from the manufacturers, specimens of all new preparations and appliances which may be brought out from time to time.]

### SPIRITINE LIMITED.

(Spiritine, Limited, 5 Carteret Street, Westminster, London, S.W.)

Spiritine is a solid spirit fuel, supplied in small tin cases with a screw top. It can be applied to all the purposes for which ordinary methylated spirit is generally used: that is to say, for sickroom and nursery cookery, for the heating of curling-tongs, and for the boiling of test-tube contents in clinical examinations of urine, &c. Being in solid form, it possesses the supreme advantage of travelling well; and, further, it cannot be upset. We have heard it well spoken of by those who put it to practical test in the Yeomanry Hospitals in South Africa. In these institutions its advantages were highly appreciated by doctors, nurses, and patients. It certainly obviates many of the dangers attendant on the use of a liquid fuel for heating purposes, and we strongly commend Spiritine to the notice of our readers.

### RAGUS SUGAR.

(Monocane Sugar Co., 13 Fish Street Hill, London, E.C.)

Ragus sugar is a pure natural sugar prepared from the Barbadoes cane. At the present day it is almost impossible to obtain any other than beet sugar from grocers and stores. Ragus sugar will be therefore welcomed by those who have a predilection for cane sugar and do not know how to obtain it. Although on scientific grounds we are unable to subscribe to the belief that cane sugar is more wholesome than that prepared from the juice of the beet, nevertheless there can be no doubt that the highly refined sugar of Continental manufacture is less agreeable to the taste and of lower sweetening properties than the pure product of the cane, such as Demerara and Barbados sugar. Householders will therefore be glad to know that by asking at their grocer's for Ragus sugar they will not only obtain a genuine, old-fashioned brown sugar, prepared solely from the cane, but that at the same time they will be supporting a most deserving and long-suffering Imperial industry.

